# Editorial: Perspectives in molecular viral pathogenesis

**DOI:** 10.3389/fcimb.2026.1797985

**Published:** 2026-02-09

**Authors:** Chun Kiat Lee, Katarzyna Otulak-Kozieł

**Affiliations:** 1 Molecular & Translational Diagnostics Laboratory, Department of Laboratory Medicine, National University Health System, Singapore, Singapore; 2 Institute of Biology, Department of Botany and Plant Physiology, Warsaw University of Life Sciences-SGGW, Warsaw, Poland

**Keywords:** cholinergic anti-inflammatory reflex, lipid raft pleomorphism, metabolic hijacking, molecular viral pathogenesis, restorative medicine, synaptic resilience, vagus-HPA-mitochondrial axis

The discipline of molecular viral pathogenesis stands at a transformative juncture in 2025. For much of the late 20th and early 21st centuries, the field was defined by a reductionist focus on the acute replication cycle (Enquist and Editors of the Journal of Virology, 2009; Fang and Casadevall, 2011). Research prioritized the “how” of viral entry, the “mechanics” of genome replication, and the “kinetics” of lytic exit. While this approach yielded the antiviral pharmacopeia currently in use, it has increasingly proven insufficient to explain the complex, multi-systemic, and chronic sequelae that characterize modern viral threats.

We are now compelled to reconceptualize the virus not merely as a transient intracellular pathogen, but as a persistent and sophisticated engineer of the host’s neuro-immuno-endocrine landscape. This shift is driven by the realization that the clinical footprint of a virus often extends years or even decades beyond the clearance of acute viremia. This reality has been brought into sharp focus by the global burden of Long COVID and mounting evidence linking viral pathogens to neurodegenerative and metabolic diseases. The articles collected in the “Perspectives in Molecular Viral Pathogenesis: 2025” Research Topic integrate these concepts, offering a cohesive picture of the virus as a master regulator of host equilibrium.

A dominant theme in contemporary virology is the persistence of physiological disruption long after the acute infectious agent is ostensibly cleared. In their cornerstone perspective, Camici et al. address the pathogenesis of Long COVID by proposing a unifying model cantered on the chronic dysfunction of the “vagus nerve-hypothalamic-pituitary-adrenal (HPA)-mitochondrial axis”. This model integrates the concept of the “vagal anti-inflammatory reflex”, which serves as the body’s primary neural mechanism for modulating systemic inflammation. The authors argue that SARS-CoV-2 infection instigates a functional “vagal neuropathy”. This is supported by empirical post-mortem evidence demonstrating direct SARS-CoV-2 infection of the vagus nerve (VN) and significant neuroinflammation within the brainstem (Woo et al., 2023). When this neural brake fails, the body loses its ability to resolve inflammation effectively. This vagal dysfunction is compounded by a maladaptive HPA axis response. Under normal physiological stress, the HPA axis triggers cortisol release to suppress cytokine production. However, Camici et al. highlight that Long COVID patients often exhibit blunted cortisol levels or glucocorticoid receptor (GR) resistance. This prevents the resolution of the inflammatory state, creating a loop of chronic cytokine dysregulation. The “anchor” of this systemic failure is the mitochondrion. As viruses hijack mitochondrial bioenergetics to fuel their own replication, it results in a persistent “bioenergetic failure” and cellular oxidative imbalance. The authors specifically point to the presence of mitochondrial and viral proteins in patient-derived exosomes as a potential driver of neuropsychiatric symptoms. Their call for diagnostic protocols evaluating GR sensitivity and epigenetic alterations offers some concrete, physiologically grounded roadmap for managing post-viral syndromes.

While Long COVID represents a post-viral syndrome measured in years, Lee et al. extend the horizon to decades, addressing the “viral-inflammatory hypothesis” of Alzheimer’s Disease (AD). Lee et al. provided a critical re-evaluation of the current dementia research landscape. They argue that the repeated failure of clinical trials targeting amyloid-beta (Aβ) stems from a fundamental misunderstanding of viral-induced neurodegeneration. Drawing on the Antimicrobial Protection Hypothesis, they suggest that amyloid plaques are likely inert “tombstones” of past successful immune defences against pathogens like HSV-1 (Soscia et al., 2010; Kumar et al., 2016). Therefore, clearing amyloid after extensive damage has occurred is unlikely to restore cognitive function. Instead, the authors advocate for a paradigm shift toward synaptic resilience. They propose that future clinical trials must utilize synaptic biomarkers to measure functional connectivity directly. By monitoring CSF-based synaptic markers such as YWHAG: NPTX2, Neurogranin (Ng), Growth-Associated Protein 43 (GAP-43), and TAR DNA-binding protein 43 (TDP-43), researchers can detect whether an antiviral or immunomodulatory therapy is successfully preserving synaptic integrity, even if the plaque burden remains unchanged. This perspective reorients the field toward preserving function rather than simply chasing the ghosts of past infections, i.e., removing pathological debris.

Viruses are obligatory metabolic pathogens that commandeer host resources to replicate. de Oliveira et al. provide an elucidation of this process in the context of male reproductive health. Using a K18-hACE2 mouse model, the authors investigated why severe COVID-19 frequently leads to low testosterone (hypogonadism). They discovered that SARS-CoV-2 exhibits a specific tropism for Leydig cells. However, the virus does not simply destroy these cells; it repurposes their lipid machinery. Infected Leydig cells show a significant upregulation of several lipid metabolism genes such as Sterol Regulatory Element-Binding Protein (SREBP), Diacylglycerol Acyltransferase 1 (DGAT-1), and Scavenger Receptor Class B Member 1 (SCARB1). This leads to a massive accumulation of lipid droplets, which the virus utilizes as physical platforms for its own assembly. This represents a “zero-sum game” for the host: the cholesterol that should be converted into testosterone via the Steroidogenic Factor 1 (SF-1) pathway is instead “stolen” to build viral components. This metabolic theft provides a precise molecular explanation for the low testosterone and HDL levels observed in severe COVID-19 patients and highlights possible therapeutic targets to prevent these effects.

The interaction between a virus and its host is, at its core, a physical event. Two articles in this Research Topic illuminate how the manipulation of cellular architecture drives pathology. Ehara et al. explored how human cytomegalovirus (HCMV) leads to secondary glaucoma. They found that HCMV infection of Human Trabecular Meshwork Cells (HTMCs), the cells responsible for draining fluid from the eye, triggers a specific chemokine cascade. The infection induces the secretion of Interleukin-8 (IL-8) and C-C Motif Chemokine Ligand 2 (CCL2). These act as signals to activate Rho GTPases, specifically Cdc42 and Rac1. The activation of Cdc42, driven by IL-8, causes the HTMCs to physically contract. This contraction increases the resistance to aqueous humor outflow, mechanically elevating intraocular pressure. This connects a viral infection directly to a mechanical tissue dysfunction. In a parallel study of viral architecture, Pastey et al. decoded the pleomorphism of respiratory syncytial virus (RSV). RSV is unique in that it buds from lipid rafts (cholesterol-rich membrane microdomains) in two distinct shapes: spherical and filamentous. The authors identified distinct molecular signatures for these forms. Filamentous particles contain significantly higher levels of uncleaved Fusion protein (F0). While spherical particles are efficient for cell-free transmission, the filamentous forms are better suited for syncytium formation, thus allowing the virus to spread directly from cell to cell while evading neutralising antibodies. This structural flexibility is a key determinant of RSV’s high virulence in pediatric and elderly populations.

The research presented in the “Perspectives in Molecular Viral Pathogenesis: 2025” Research Topic paints a comprehensive picture of viruses as sophisticated manipulators of host biology. While these contributions span the spectrum from structural biology to systemic physiology, a cohesive narrative emerges: clinical disease is not merely the result of viral presence, but the manifestation of host machinery being coerced into a maladaptive state. Pastey et al. demonstrate how RSV manipulates host lipids for pleomorphic assembly, a theme echoed by Ehara et al. in the context of ocular pathology, where molecular signals (IL-8 and CCL2) translate into mechanical cell contraction and secondary glaucoma. de Oliveira et al. reveal the specific metabolic cost of this invasion within the male reproductive system, showing how viral resource “theft” in Leydig cells leads to significant testosterone deficiency and impaired steroidogenesis. Furthermore, the Research Topic is rounded out by the work of Lee et al. and Camici et al., who illustrate the long-term consequences of these molecular insults. They describe how viral disruption of neuro-immune and neuro-endocrine axes can spiral into chronic, multi-systemic syndromes like AD and Long COVID, respectively.

As we move forward, the integration of these molecular insights with clinical observation will be essential for developing therapies that transcend simple viral suppression. Virology must evolve toward restorative medicine, seeking not only to clear pathogens but to actively recalibrate disrupted neuro-endocrine axes, fortify the functional synaptic architecture, and reclaim the metabolic assets commandeered during infection. By shifting from a purely lytic view of infection toward a systems-biology perspective, we can begin to address the whole-body equilibrium that viruses so effectively destabilize. This paradigm shift promises a new era of precision medicine, where the clinical objective is not merely to eliminate the invader, but to repair and restore the complex biological architecture that remains its primary target.
